# Spectral exponent assessment and neurofilament light chain: a comprehensive approach to describe recovery patterns in stroke

**DOI:** 10.3389/fneur.2024.1329044

**Published:** 2024-03-18

**Authors:** Jacopo Lanzone, Aida Zulueta, Marilisa Boscarino, Matteo Gallotta, Maria Rosaria Argentieri, Alessandro Viganò, Simone Sarasso, Michele A. Colombo, Sasha D’Ambrosio, Christian Lunetta, Eugenio Parati

**Affiliations:** ^1^Istituti Clinici Scientifici Maugeri IRCCS, Neurorehabilitation Department of the Milano Institute, Milan, Italy; ^2^IRCCS Fondazione Don Carlo Gnocchi, ONLUS, Milan, Italy; ^3^Department of Biomedical and Clinical Sciences, Università Degli Studi di Milano, Milan, Italy; ^4^Department of Health Sciences, Università Degli Studi di Milano, Milan, Italy; ^5^Department of Clinical and Experimental Epilepsy, University College London, London, United Kingdom

**Keywords:** stroke, rehabilitation, EEG, neurofilament, biomarkers

## Abstract

**Introduction:**

Understanding the residual recovery potential in stroke patients is crucial for tailoring effective neurorehabilitation program**s.** We propose using EEG and plasmatic Neurofilament light chain (NfL) levels as a model to depict longitudinal patterns of stroke recovery.

**Methods:**

We enrolled 13 patients (4 female, mean age 74.7 ± 8.8) who underwent stroke in the previous month and were hospitalized for 2-months rehabilitation. Patients underwent blood withdrawal, clinical evaluation and high-definition EEG at T1 (first week of rehabilitation) and at T2 (53 ± 10 days after). We assessed the levels of NfL and we analyzed the EEG signal extracting Spectral Exponent (SE) values. We compared our variables between the two timepoint and between cortical and non-cortical strokes.

**Results:**

We found a significant difference in the symmetry of SE values between cortical and non-cortical stroke at both T1 (*p* = 0.005) and T2 (*p* = 0.01). SE in the affected hemisphere showed significantly steeper values at T1 when compared with T2 (*p* = 0.001). EEG measures were consistently related to clinical scores, while NfL at T1 was related to the volume of ischemic lesions (*r* = 0.75; *p* = 0.003). Additionally, the combined use of NfL and SE indicated varying trends in longitudinal clinical recovery.

**Conclusion:**

We present proof of concept of a promising approach for the characterization of different recovery patterns in stroke patients.

## Introduction

The management of acute stroke has advanced considerably over the last decade. Treatments such as systemic thrombolysis or mechanical thrombectomy are becoming a standard of care, and prompt reperfusion is now available for many patients. Likewise, as the population ages and acute treatments improve, the prevalence of stroke-related disabilities is on the rise (1).

Rehabilitation stands as the primary means to minimize disability and facilitate the return of stroke patients to their optimal functioning within their home environment (2). However, evidence on stroke rehabilitation shows considerable inter-individual variability in functional recovery (3). Most patients seem to follow a proportional model of recovery. Nevertheless, there are several cases in which this model does not apply (4).

One of the main issues in describing recovery after a stroke is our predominant reliance on clinical scales that gauge residual functionality (5). This approach could potentially be biased, as clinical scales alone may fail to capture crucial underlying processes like plasticity, functional reserve, or hemispheric interactions (6). When two patients, matched for age and comorbidities, exhibit identical clinical scores shortly after experiencing a stroke, significant variations in their paths to recovery tend to emerge as they transition into the chronic phase. These differences are believed to be associated with essential factors that go beyond clinical assessments and rely on the brain’s functional and structural resilience.

Furthermore, according to the bimodal balance recovery model proposed by Di Pino et al. (7, 8), ischemic lesions alter the balance between hemispheres creating two possible scenarios. The first is where residual activity in the affected hemisphere is found and plasticity from the unaffected hemisphere could hinder recovery. The latter is where there is no residual activity in the affected hemisphere thus necessitating the unaffected hemisphere to assume a pivotal role in compensating for the loss of function.

The bimodal recovery model could partly explain the inhomogeneous rehabilitative outcomes that we observe post-stroke. Eventually, different scenarios could need different interventions. Hence, we need instruments that allow us to better characterize patients.

Our objective is to identify comprehensive measures that characterize longitudinal recovery patterns in stroke patients. To facilitate their implementation across a broad patient population, these measures are supposed to be both user-friendly and easily scalable. Reliable biomarkers of stroke recovery could help us identify patients with greater rehabilitative potential and develop tailored approaches in neurorehabilitation.

Neurophysiology offers a wide array of tools that can inform prognosis in stroke patients (9). EEG, in particular, has been studied thoroughly in both acute and chronic stroke (10).

We recently evidenced how the Spectral Exponent (SE) of the EEG is a powerful neurophysiological fingerprint of stroke (11). The EEG SE describes the 1/f-like structure of the Power Spectral Sensity (PSD) (12) and is consistently steeper in the Affected Hemisphere (AH), a finding also confirmed in animal models (13).

We use EEG measures to assess recovery in stroke patients because these markers reflect the functional state of brain macroscale activity with high temporal definition. However, quantitative EEG metrics do not provide direct structural damage entity information. For instance, even relatively small lesions can cause widespread EEG slowing (14). This can happen due to the impact of the lesion on intact but functionally connected areas (15), a phenomenon also known as diaschisis (16).

To overcome the bias of using “mono-dimensional” measures, an approach based on the combination of different biomarkers has been proposed (17).

We hypothesize that the simultaneous use of EEG measures and markers of brain structural damage could offer distinctive insights into the post-stroke recovery pattern, transcending the limitations of each method by itself.

Single Molecule Array (SiMoA) assays enable the measurement of neurological biomarkers related to brain injury (18). This novel tool opened an interesting debate on the role of biomarkers and how they relate to the process of recovery (19, 20). Among these, neurofilaments have gained increasing attention since they are structural proteins exclusively expressed in neurons that are released because of axonal damage, not only in cerebrospinal fluid, but also in peripheral blood in many neuropathological conditions (21). Thus, we identified neurofilament light chain (NfL), as a biomarker that could complement EEG in the longitudinal assessment of stroke recovery.

In this paper, we propose a new simple model of longitudinal characterization of patients recovering from a stroke, leveraging neurophysiological measures (EEG) and biological markers of neural damage (NfL). These two instruments can be easily employed, are cost and time effective, and thus are suited to be employed in clinical longitudinal follow-up.

We present this work as a proof-of concept. In the future, an up-scale model could let us describe clusters of longitudinal recovery in stroke and develop tailored approaches to rehabilitation for different clusters of patients.

## Methods

### Patient enrollment

Thirteen patients affected by ischemic stroke were consecutively enrolled at ICS Maugeri institute of Milan. Enrolment period was between March 2022 and March 2023. Patients, suffering from acute stroke, were admitted at our center to undergo 60 days post-stroke intensive rehabilitation cycle provided by the Italian National Health System.

We used the following inclusion and exclusion criteria:

#### Inclusion criteria

(I) Mono hemispheric ischemic stroke within the last month, (II) supra-tentorial lesion, (III) Ability to undergo clinical assessment and EEG recording.

#### Exclusion criteria

(I) Previous large stroke, (II) Other previous or concomitant disorders with central nervous system involvement (Parkinson’s disease, Alzheimer, brain tumor, chronic subdural hemorrhage), (III) Multifocal lesions, (IV) Large hemorrhagic lesions, (V) longitudinal recording and blood withdrawal outside of the declared time limits. (VI) failure to obtain good EEG and biological data.

The local ethical committee of IRCCS Fondazione Maugeri approved the experimental protocol and each patient gave written informed consent to participate in this study. Data was pseudonymized and analyzed in accordance with the current General Data Protection Regulation (GDPR) guidelines.

### Rehabilitation protocol

Patients enrolled followed a rehabilitation protocol consisting of 1 h a day, 6 days a week of physical therapy (1 on 1 work with the physiotherapist) and an additional 1 h divided between logotherapy and occupational therapy. This protocol follows the standards of what is provided by our public health care system in cases of stroke.

### Clinical assessment

We collected demographic and clinical data regarding acute stroke and clinical interventions (I.e., thrombolysis), as summarized in Table 1. Patients were evaluated by experienced clinicians using the National Health Institute Stroke Scale (NIHSS) (22) and the Functional Independence Measure (FIM) (23), which are among the most frequently used outcomes measures in stroke rehabilitation, allowing to assess neurological improvement (NIHSS) and residual disability (FIM). Clinical evaluation was performed at T1 (within the first week from admission), and at T2 (after about 2 months of). Clinical scores were obtained during the same day of blood withdrawal and EEG recording. Additionally, we derived the Effective Recovery rate (ER) as the percentage improvement in NIHSS (NIHSST1-NIHSST2/NIHSST1), frequently used as a measure of longitudinal evolution.

**Table 1:** Clinical and demographic data.

ID	SIDE	VOLUME	CORT	NIHSS_T1	NIHSS_T2	ER	FIM_T1	FIM_T2	NF-L_T1	NF-L_T2
1	L	3.32	1	4	2	50.0	64	77	407.70	197.98
2	R	24.88	1	9	4	55.6	56	62	1426.75	216.01
3	L	4.35	0	8	5	37.5	57	83	221.72	155.09
4	L	0.43	0	9	5	44.4	21	57	148.85	66.76
5	R	3.84	0	8	4	50.0	57	112	262.24	149.94
6	L	14.77	1	16	11	31.3	23	52	612.19	247.29
7	R	1.54	0	5	1	80.0	107	122	190.88	188.94
8	R	79.25	1	9	7	22.2	79	98	1698.85	675.30
9	R	0.41	0	7	4	42.9	90	117	216.98	27.09
10	R	12.24	1	5	1	80.0	99	116	1027.57	354.13
11	L	101.36	1	20	18	10.0	37	41	1156.34	616.96
12	L	0.55	0	3	1	66.7	101	103	637.90	190.48
13	L	4.16	1	7	3	57.1	73	83	144.52	77.57

NIHSS (National Institute of Health Stroke Scale), ER (Effective recovery Rate), FIM (Functional Independence Measure), NfL (Neuro Filament Light chain); CORT (cortical, yes = 1); L = left; R = right, T1 (First timepoint), T2 (Second timepoint).

### Imaging

Patients underwent a standard CT head scan at T1 to estimate lesion volume, location and define cortical involvement (cortical and non-cortical), scans were reviewed by an experienced neurologist and lesion volumes were manually marked for each slice using MRIcron software (24). Then, the 3D region of interest was visually checked (representative images of lesion localization for each patient are presented in Supplementary Figure S1).

### Plasma sample collection

Blood samples from each patient were collected at T1 and at T2 in tubes containing ethylenediaminetetraacetic acid (EDTA) as anticoagulant and centrifuged at 2,000 × g for 15 min at room temperature. Plasma was aliquoted and stored at −80°C until use.

### Analysis of plasma neurofilament light chain levels

Quantitative analysis of NfL in plasma samples of patients was performed by single molecule array (SiMoA) technology on the SR-X analyzer from Quanterix (Billerica, MA, United States). NfL levels were determined using the commercially available Simoa NFLIGHT v2 Advantage kit (Item 104,073, Quanterix), according to the manufacturer’s instructions. Briefly, samples, calibrators and two quality controls of known concentrations (high-concentration and low-concentration quality control) were run in duplicate. Samples were run with a 4-fold dilution and results were compensated for this dilution. The mean value of the two NfL measurements (pg/ml) was used for statistical analysis. The limit of quantification was 2.56 pg/mL, and the limit of detection was 0.141 pg/mL. A single batch of reagents was used for all samples; and the intra-assay coefficient of variation was below 14%.

### EEG recordings

Patients underwent resting eyes-closed EEG recording at T1 (within the first week of admission), and at T2 (during the last week of rehabilitation). EEG recordings were performed by means of a 60-channel amplifier (Nexstim Ltd.) with associated pre-wired head-cap. Raw signals from each of the 60 recording channels were sampled at 1450 Hz, referenced to the forehead and filtered (hardware filters were set at 0.1 Hz and 350 Hz). EEGs were recorded in a quiet room with dim lights, with the patient sitting in a comfortable armchair. After ensuring that all channels had an impedance below 5KΩ, 5 min of EEG were recorded. EEG recording started after the patient was calm and adapted to the seat and recording condition to minimize artifacts. The vigilance and wake state of the patients was continuously monitored by EEG inspection to exclude sleepiness or drowsiness. Data was exported in digital format for further analysis.

### EEG signal analysis

EEG recordings were analyzed using MATLAB© native code and using code from the EEGlab toolbox (25). EEG analysis focused on eliminating non-brain artefactual activity as done in previous studies (11, 26). The time-series were detrended with respect to long-range linear trends and filtered with an IIR high-pass (5th order Butterworth filter with a 0.5 Hz cut-off) and a notch filter centered at 50 Hz. Bad channels and timepoints were manually selected, with the aid of a custom informative system that displays and quantifies large fluctuations over time-points and channels. Bad channels were interpolated (spline interpolation). Channels were then re-referenced to average reference. Independent Component Analysis (ICA) was performed, and non-brain components were manually rejected with the aid of a custom informative system, that displays and quantifies relevant properties of the components, based on temporal, spectral and spatial features, tailored to characterize muscular, ocular, and cardiac artifacts.

The Power Spectral Density (PSD) was estimated using Welch’s method (2 s window, 50% overlap). The Spectral Exponent (SE) of the 1–20 Hz range was estimated for each pre-processed EEG channel. The code to calculate the SE is openly available online^1^ and the specific methods thoroughly described explained in (26). Briefly, the spectral exponent reflects the decay rate of the PSD over increasing frequencies, and is thus a measure of slowing, measured over a broad frequency range. We then computed, for the SE values, the mean among the channels of the Affected Hemisphere (AH) and of the Unaffected Hemisphere (UH), and the ratio between the average value of AH channels divided by the average value of the UH channels (AH/UH ratio), since metrics of signal asymmetry are typically used in quantitative EEG analysis, especially in stroke research (27, 28).

### Statistical analysis

Data was imported in R studio; descriptive statistics are presented for clinical and demographic variables. We used Wilcoxon-signed-rank test to assess longitudinal changes (T1 vs. T2) in NfL, clinical, and EEG variables. We calculated the (Spearmam) correlation among variables of interest, and used a heatmap correlogram, ordered according to hierarchical clustering, to visually depict the correlations in our data set. Mann–Whitney test was used to assess the difference between the cortical and non-cortical lesion groups. For all statistical comparisons, the alpha level of significance was set at 0.05. Results are presented as adjusted *p* values after multiple comparison correction False Discovery Rate (FDR), across all pairs of markers and correlation analysis. The full study protocol is depicted in Supplementary Figure S2.

## Results

### Demographic

Thirteen patients (74.7 ± 8.8 years old, 4 females) completed the experimental protocol. The average time from acute stroke was 20.2 ± 6 days, the average distance between T1 and T2 was 53 ± 10 days. Eight patients were classified as Anterior Circulation Stroke (ACS), four as Lacunar Cerebral Infarction (LACI). Seven patients were classified as patients with cortical involvement according to CT scans and/or MRI acquired in the acute setting. Two patients underwent systemic thrombolysis, none was treated with mechanical thrombectomy. Only one patient presented minimal hematic spotting at CT scan.

### Clinical scores

There was a significant (Z = –3.14, *p* = 0.001) improvement in NIHSS scores from T1 (median 8, IQR 5|9) to T2 (median 4, IQR 2|5). Likewise, FIM scale both the motor sub score (FIM_M, T1: median 45, IQR 22|55, T2: median 56, IQR 54|72, Z = –3.66, *p* < 0,001) and global score (FIM; T1: median 64, IQR 56|90, T2: median 83, IQR 62|112, Z = –3.68, *p* < 0,001) showed significant longitudinal improvement. The percentage of measured clinical improvement was 48.2 ± 18% for NIHSS. No substantial differences in FIM and NIHSS were observed between patients with and without cortical lesions at either of the two time points.

### Correlation analysis

**Figure 1:**
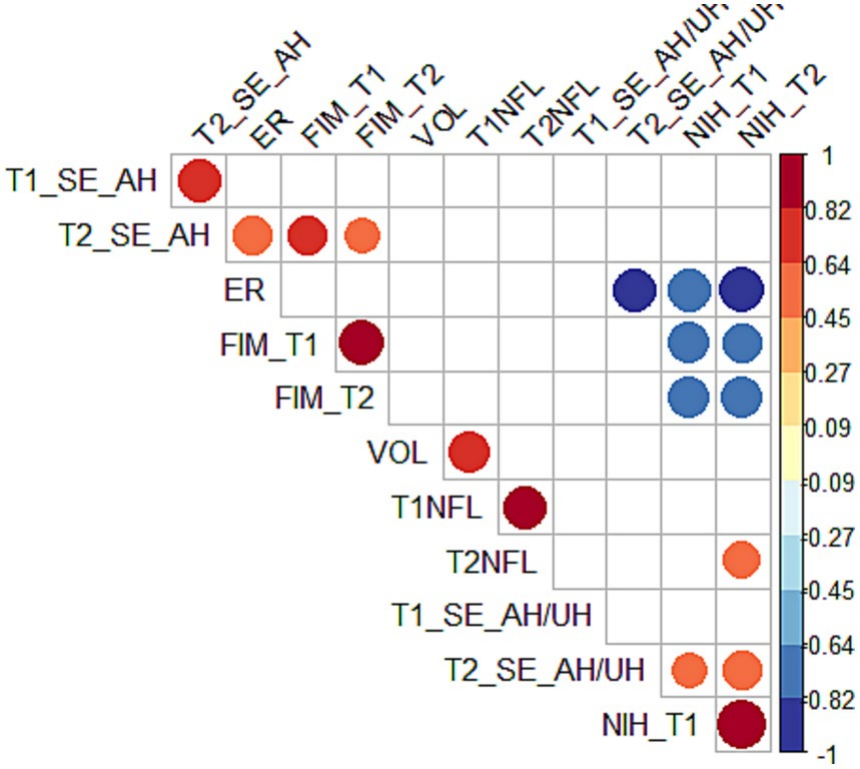
Correlation between outcome variables and EEG and plasmatic biomarkers. Here we show the correlation matrix highlighting significant correlations in our dataset. Pairs are grouped according to hierarchical clustering, so that variables that show similar patterns are clustered together. NIH (National Institute of Health Stroke Scale), ER (Effective recovery Rate), FIM (Functional Independence Measure), NfL (Neurofilament Light chain); AH/UH (ratio between Affected [AH] and unaffected hemisphere [UH] metrics); SE (Spectral Exponent), T1 (First timepoint), T2 (Second timepoint).

In Figure 1 we show the correlation map of our main variables organized according to hierarchical clustering. We found significant longitudinal and cross correlations among clinical variables (i.e., FIM and NIHSS at T1 and T2). This was expected since clinical scores all depend on the same latent construct, the same can be said for different EEG metrics. Interestingly, we found a significant correlation between EEG metrics and NIHSS. Namely between NIHSS_T2 and T2_AH/UH_SE (r = 0.57; *p* = 0.04), NIHSS_T1 and T2_AH/UH_SE (*r* = 0.55; *p* = 0.05); ER and T2_AH/UH_SE (*r* = 0.67; *p* = 0.001); ER and T2_SE_AH (*r* = −0.67; p = 0.001); FIM_T1 and T2_SE_AH (*r* = 0.56; p = 0.05); FIM_T2 and T2_SE_AH (*r* = 0.66; p = 0.04). These correlations show that asymmetrical EEG activity with a steeper slope in the AH relates to a lower percentage of recovery (ER) and higher NIHSS at both timepoints.

Lesion volume strongly correlated to NfL levels at T1 (*r* = 0.75; *p*==0.003). Finally only NfL at T2 showed correlation with NIHSS scores at T2 (*r* = 0.57; *p*==0.03).

### Group comparisons

#### T1 Vs. T2 differences in EEG metrics and NfL

We observed a strong significant (*Z* = -3.67, *p* < 0.001) decrease in NfL plasmatic level from T1 (median = 407.7, IQR 216|1,027 pg./mL) to T2 (median = 190.5, IQR 145|247 pg./mL). The longitudinal difference between SE was significant for the AH (*Z* = -2.47, *p* = 0.001; T1 median − 1.154 IQR –1.01|-1.35; T2 median − 1.009 IQR –0.85|-1.23), but not for the UH. However, AH/UH ratio of SE showed a trend toward significance comparing T1 and T2 (*Z* = -1.824, *p* = 0.07).

### AH-UH differences

As previously reported (11), we confirmed differences between AH (Median SE = -1.154 IQR –1.01|-1.35) and UH SE (Median SE = -1.012, IQR –0.88|-1.11) at T1 (*Z* = -2.47; *p* = 0.015). On the other hand, no interhemispheric differences were found at T2.

### Differences between cortical and non-cortical lesions

We tested differences in outcome measures between the cortical and non-cortical groups. We observed a significant difference in T1 NfL (cortical: median 1,027, IQR 509|1,291 pg./mL, non-cortical: median 219, IQR 197|252 pg./mL, Z = -1.86 *p* = 0.05) and T2 NfL (cortical: median 247, IQR 206|485 pg./mL, non-cortical: median 152, IQR 87|180 pg./mL, *Z* = 2.51; *p* = 0.02). Furthermore, we observed a significant difference in T1_AH/UH_SE (cortical: median 1.19, IQR 1.15|1.57, non-cortical: median 1.006, IQR 0.97|1.03 pg./mL, *Z* = -2.82; *p* = 0.005) but not in T2_AH/UH_SE. There were no significant differences in SE AH (T1 or T2) values between cortical and non-cortical strokes. Finally, there were also significant differences in lesion volume (cortical: median 14.77, IQR 8|72 cc, non-cortical: median 1.04, IQR 0.46|3.26 cc, *Z* = -2.46; *p* = 0.01).

## Discussion

**Figure 2:**
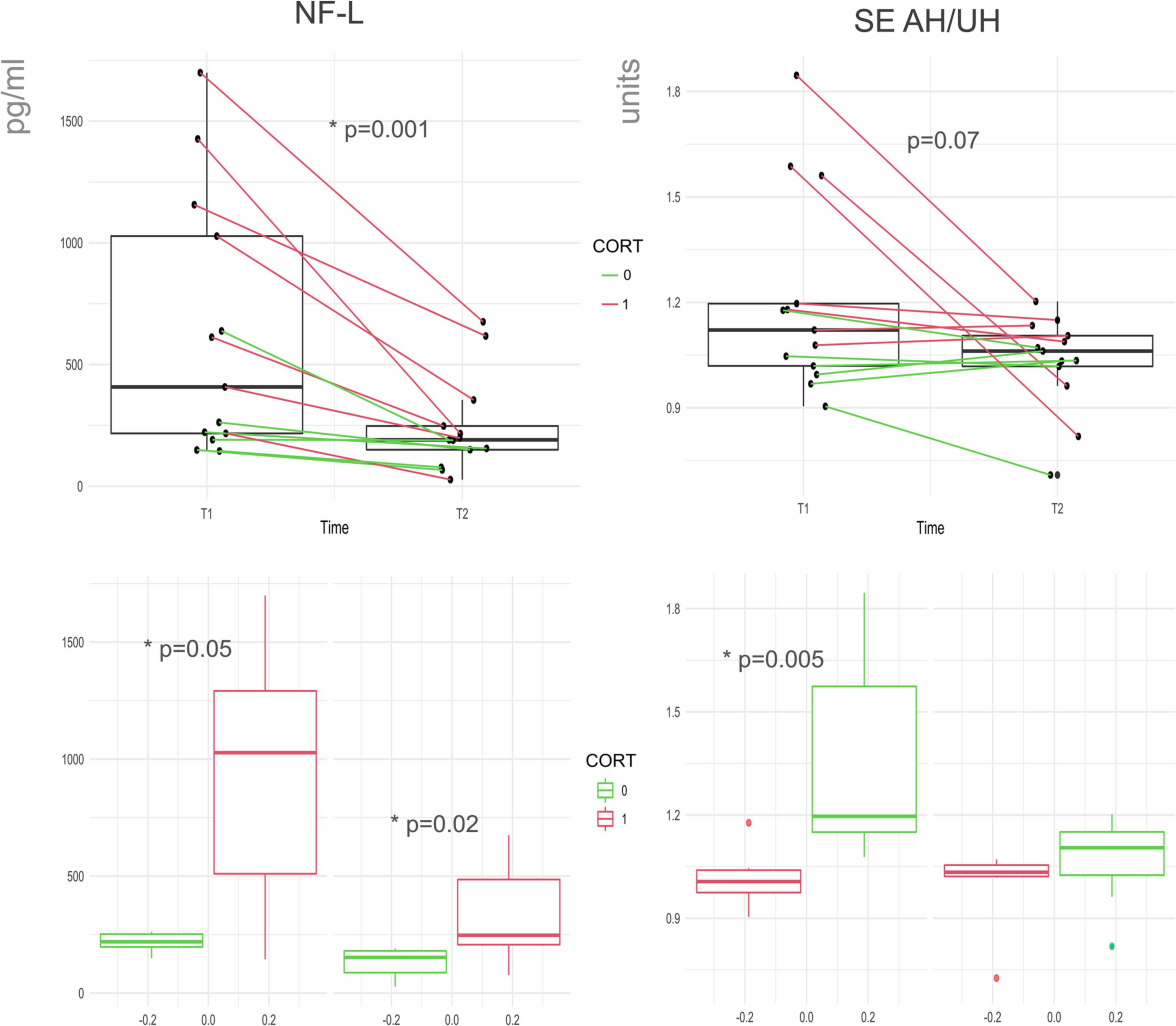
NfL and SE change over time and according to cortical involvement. Here we evidence changes across the two timepoints (T1 and T2) in NfL and SE AH/UH ratio, consecutive recordings from the same patient are linked. Cortical (red) and non-cortical groups (green). Below we show the group differences between patients with and without cortical involvement for NfL and SE at both timepoints. NfL (Neuro Filament Light chain); AH/UH (ratio between Affected and non-affected hemisphere metrics); SE (Spectral Exponent).

**Figure 3:**
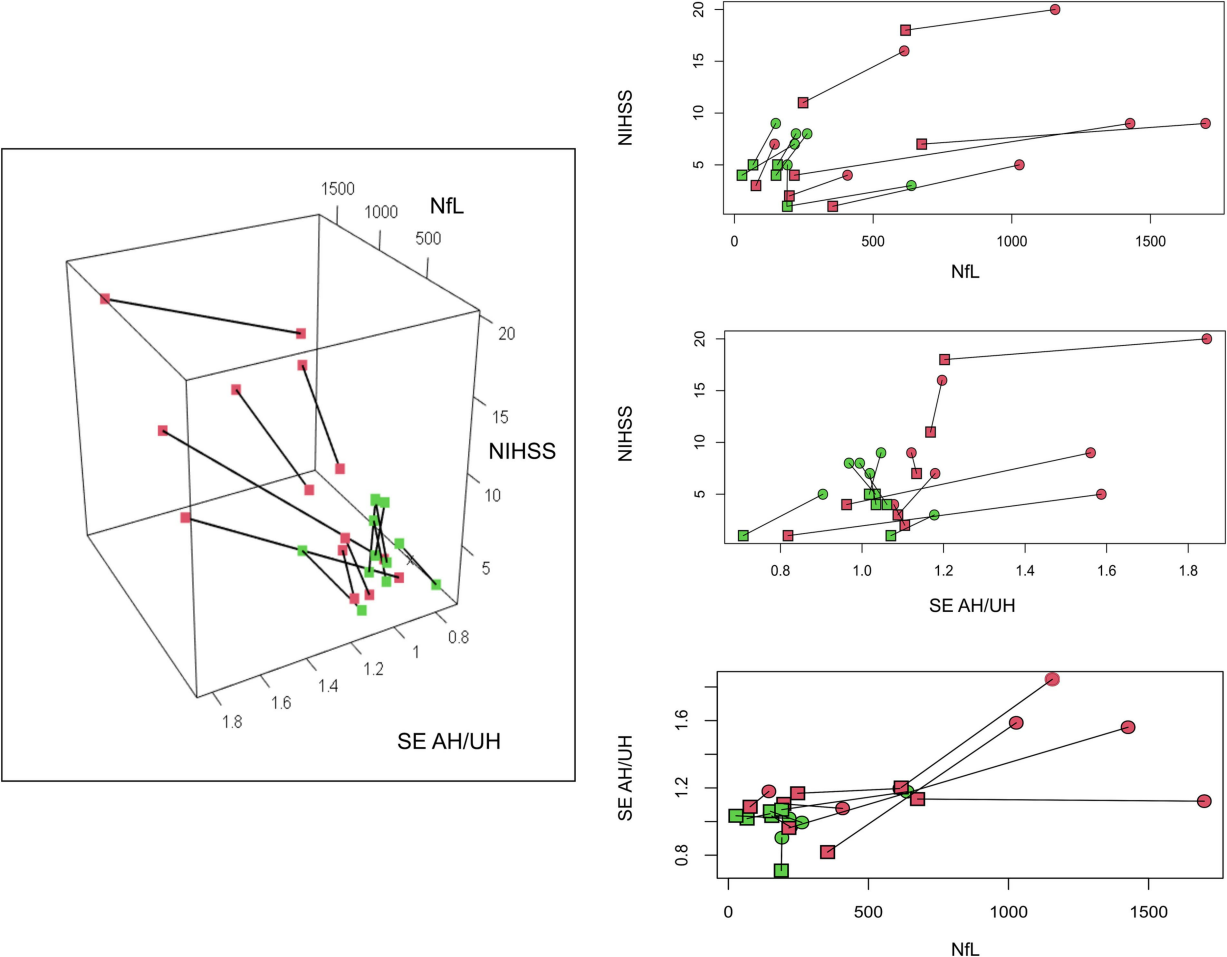
Different longitudinal recovery pattern. Here we show how EEG and Nf-L interact with the clinical status of our patients group, using a a 3-dimensional plot. The location of the lesion in our patients is color-coded (red cortical, green non-cortical). For ease of consultation, we presented the corresponding two-dimensional plots. T1 (box) and T2 (dot) recordings are linked. NfL (Neuro Filament Light chain); AH/UH (ratio between Affected and non-affected hemisphere metrics); SE (Spectral Exponent), NIHSS (National Institute of Health Stroke Scale).

In our study we show fundamental differences between cortical and non-cortical strokes, both in NfL and in EEG markers (Figures 2, 3). This highlights the capability of our approach to distinguish between two important categories of stroke. Stroke with cortical involvement is known to present marked EEG slowing (29), while small subcortical lesions not involving large white matter areas, such as lacunar stroke, do not seem to have a deep impact on EEG rhythms.

In alignment with our prior findings (11), we have reaffirmed the functional significance of EEG measures and their correlation with clinical scores, as depicted in Figure 1. As previously elucidated, this clinical correlation becomes particularly conspicuous at the T2 time-point, in the chronic phase. This finding is likely attributed to residual lesional hemorrhage, edema and inflammation at T1. Such transient phenomena could contribute to overestimating NfL and EEG parameters in the acute to sub-acute phases. In contrast, lesion volume does not exhibit correlation with clinical scales, while NfL showed only marginal correlation at T2. As we know from clinical experience, larger brain lesions do not necessarily translate to more significant clinical impairment.

Our results on NfL show a clear and univocal reduction over time, supporting the evidence that NfL can be a marker of lesion evolution during the post-acute stroke phase. This was already proposed but-to the best of our knowledge-previous literature lacked paired longitudinal controls or did not find a clear and univocal NfL reduction over time (18, 30–32). Additionally, we further confirmed the relationship between lesion volume and plasmatic NfL levels, already suggested by other authors (18, 33). We also found that NfL levels were higher in cortical lesions, which are typically larger in volume. Thus, NfL is confirmed to be a reliable plasmatic biomarker of structural damage in stroke, mostly related to lesion volume and decay over time.

Regarding quantitative EEG measures, we confirmed our previous findings (34), showing significant hemispheric SE asymmetry at T1 and a reduction of AH SE values from T1 to T2. Likewise, SE showed a marginally significant reduction in hemispheric asymmetry of from T1 to T2. Our results are mainly guided by the steep values of SE found in the AH of cortical strokes, thus we also derived SE asymmetry indexes (AH/UH) to normalize data between cortical and non-cortical stroke and explore deeper the potential clinical correlation.

In conclusion, SE and NfL reflect different and additive features of the recovery process; the first grounded in macro-scale neurophysiological activity, the latter related to lesion volume and time from stroke. The combination of these biomarkers could allow an in-depth depiction of longitudinal recovery. To support this proof of concept, in Figure 3 we summarized the relation between NIHSS, EEG and NfL. It can be seen how cortical stroke and non-cortical stroke can be clearly distinguished. Even if they have similar improvement in NIHSS scores, cortical stroke shows more marked changes over time in NfL and SE. We also notice that within the cortical group there is more heterogeneity.

Our approach is modular and can be improved in many ways, while keeping its simple setup. A wide variety of molecular biomarkers could be studied as clinical predictors of stroke recovery, such as biomarkers associated with inflammation, tissue remodeling, regeneration and neuronal plasticity (35, 36). At the same time, EEG connectivity could add an interesting dimension to this depiction of stroke recovery, especially for non-cortical stroke (34).

This model proposes the use of combined EEG and plasmatic biomarkers to differentiate patients that otherwise could appear similar. As suggested by the bimodal balance recovery model different patients could benefit from tailored protocols of non-invasive brain stimulation (37). In our model, we merge the neurophysiological information gained from EEG with information linked to the volume of lesioned brain tissue. In clinical neurology, it is crucial to be able to link these factors as often large lesions create small functional impairment, and *vice-versa* small lesions can be bothersome.

The following are some use cases of our proposal, that highlight how the combination of functional (EEG) and structural (NfL) information can be useful.

A patient with a thalamic lesion could present low NfL levels but lateralized alteration of EEG alteration, suggesting that he is a good candidate for neuromodulation via inhibition of the unaffected hemisphere or stimulation of the affected hemisphere to foster residual potential. In another scenario, a patient with cortical stroke could present sever EEG asymmetry and high NfL levels, suggesting that the damage is too extensive to try to develop plasticity in the affected hemisphere, and the patient could benefit from excitatory stimulation of the unaffected hemisphere to develop plasticity.

In the future, multidimensional assessment could show that some patients with important neurophysiological alterations might therefore benefit from non-invasive neurostimulation. On the other hand, patients with high levels of NfL or neuroinflammation could benefit from targeted therapy such as neuroprotective drugs (38).

We presented this paper as proof of concept, hoping to grow our model in complexity and numbers soon.

## Limitations

A significant constraint of our study pertains to the relatively small sample size, which is attributable to the longitudinal nature of our research and the extended duration of observation. We acknowledge that a larger sample size is needed to draw definitive conclusions, however our preliminary results are promising and novel. Furthermore, we measured lesion volume via CT, this approach gives an approximate estimate of volumes, but is likely to underestimate lesions’ volume. Finally, a denser sampling with more time points could better definition of each patient’s longitudinal trajectory and inform us on the reliability of our biomarkers.

## Conclusion

In this paper we show proof of concept that a multimodal approach, considering both molecular and neurophysiological parameters, is feasible and informative, giving insight into the longitudinal trajectories of patients recovering from stroke. In the future this model could be used to guide tailored rehabilitation.

## Data availability statement

The raw data supporting the conclusions of this article will be made available by the authors, without undue reservation.

## Ethics statement

The studies involving humans were approved by Local ethical committee of ICS IRCCS Maugeri Pavia. The studies were conducted in accordance with the local legislation and institutional requirements. The participants provided their written informed consent to participate in this study.

## Author contributions

JL: Writing – review & editing, Writing – original draft, Supervision, Methodology, Investigation, Conceptualization. AZ: Writing – review & editing, Methodology, Data curation. MB: Writing – review & editing, Investigation. MG: Writing – review & editing, Investigation, Data curation. MA: Writing – review & editing, Investigation. AV: Writing – review & editing, Writing – original draft. SS: Writing – review & editing, Resources, Funding acquisition. MC: Writing – review & editing, Methodology. SD’A: Writing – review & editing, Supervision, Investigation, Data curation. CL: Writing – review & editing, Supervision. EP: Writing – review & editing, Supervision.

## Footnotes

^1^ https://github.com/milecombo/spectralExponent
