# Supplementary figures and images for: Spectral exponent assessment and neurofilament light chain: a comprehensive approach to describe recovery patterns in stroke
Source: Front Neurol. 2024 Mar 18;15:1329044. doi: 10.3389/fneur.2024.1329044 (PMC10982436; doi:10.3389/fneur.2024.1329044)

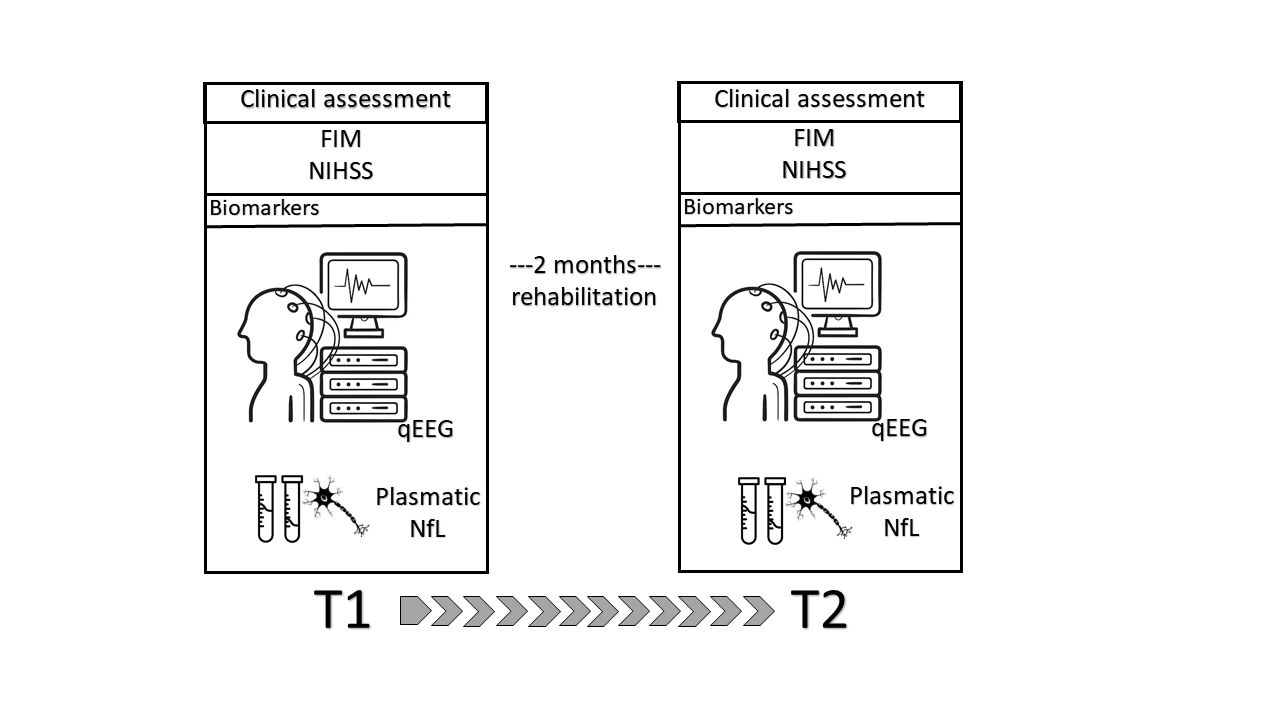

Supplement: Supplementary file 1 [file Image_1.TIF]
